# Patient safety culture in Palestine: university hospital nurses’ perspectives

**DOI:** 10.1186/s12912-022-00987-y

**Published:** 2022-07-28

**Authors:** Loai M. Zabin, Rasha S. Abu Zaitoun, Abdullah A. Abdullah

**Affiliations:** 1 Department of Nursing, An-Najah National University Hospital, Nablus, 44839 Palestine; 2 Quality and Patient Safety Department, An-Najah National University Hospital, Nablus, 44839 Palestine

**Keywords:** Patient safety culture, Hospital, Patient safety, Palestine

## Abstract

**Background:**

Understanding the perspectives of healthcare workers toward patient safety-related activities is critical in maintaining a healthy safety climate. The objectives of this research are 1) to examine the perception of Patient Safety Culture (PSC) at a university hospital in Palestine, and to highlight areas in need of improvement, and 2) to assess the relationship between the outcome dimensions (frequency of events reported, and overall perceptions of safety) and the other dimensions of PSC, and 3) to determine the relationship among selected demographic variables (gender, age, hospital tenure, work tenure, profession tenure, and hours worked per week) and nurses’ perceptions of PSC.

**Methods:**

A cross-sectional study design was used with a convenience sample of 107 nurses. Nurses were asked by email to complete the Arabic version of the Hospital Survey of Patients’ Safety Culture (HSOPSC) using the SurveyMonkey® online account form within two weeks. The survey data were analyzed using descriptive and inferential statistics. Univariate and multiple regression were used to examine the relationships.

**Results:**

The dimensions of patient safety with the highest positive response were organizational learning and continuous improvement (87%) and teamwork within units (86%). The dimension with the lowest positive score was the nonpunitive response to error (22%). Multiple regression revealed that the dimension of communication openness was a predictor of the overall perceptions of safety (β = 0.257, *p* = 0.019). In addition, the dimension of feedback and communication about error was a predictor of the frequency of the reported events (β = 0.334, *p* = 0.005). Furthermore, age was found to be a predictor of PSC (*p* < 0.05).

**Conclusions:**

This study provides a general assessment of perceived safety among nurses in a hospital. However, we found that nurses negatively perceive a nonpunitive response to error. Therefore, strenuous efforts are required by hospital management to improve the culture of incident reporting.

## Background

“To Err is Human: Building a Safer Health System” report highlighted the need to establish a safety culture in healthcare organizations and emphasized that the majority of errors occurring in the healthcare context are almost system-related; this implies the need to improve system-related issues more than individual issues [1]. Healthcare institutions internationally are trying hard to enhance the safety of patients by creating a positive patient safety culture, which is required by Joint Commission International Accreditation (JCIA) [2].

Patient safety culture (PSC) is a component of an organizational environment directly related to patient safety values and beliefs within healthcare systems [3]. Understanding the perspectives of healthcare workers toward patient safety-related activities is critical in maintaining a healthy safety climate [4]. This knowledge is important for policymakers and managers to improve patient safety [5].

Many strategies and initiatives, such as accreditations and the Patient Safety Friendly Hospital Initiative (PSFHI) had been implemented to improve PSC [6]. Recently, patient safety has become the key to hospitals’ success in Palestine, and more hospitals are applying for the JCIA. An-Najah National University Hospital (NNUH) has been recently accredited. Moreover, the Ministry of Health and NNUH joined the Initiative of the WHO’s Patient Safety Friendly Hospital.

Nurses account for about 50% of global healthcare personnel [7]. In Palestine, they are the majority and the backbone of the health care system [8]. Nurses spend most of their time with their patients, and they are essential in any healthcare organization to ensure patient safety [9]. However, the lack of personnel, work overload, teamwork behavior, underreporting of adverse events, and the lack of continuing education of professionals influence the emergence of adverse events, thus contributing to a culture where mistakes can occur and injure patients [10].

In Palestine, PSC is a recent trend, as some hospitals started enrolling in Joint Commission International Accreditation (JCIA). Nurses at NNUH in which this study has taken part are the key to this accreditation’s success with much effort, especially since it was their first experience participating in such accreditation as there is no national accreditation system in this country. Focusing on the six strategic International Patient Safety Goals (IPSG) of JCIA is a challenge, and nurses must apply new policies and procedures to ensure patients are safe, as there is a strong relationship between IPSG application and PSC [11]. Many studies had shown that patient safety is a challenge for nurses [12]. Therefore, it will help identify the issues that need to be addressed to enhance healthcare quality by identifying the factors that can affect patient safety from the nurses’ point of view at this hospital.

Because measuring PSC could aid healthcare institutions in recognizing fields for enhancement and tracking changes efficiently over time [13], this study investigated the perceptions of PSC from the nurses’ view of a university hospital in Palestine. The ultimate goal is to identify areas for improvement as well as to provide a baseline for evaluating potential improvement initiatives in the future.

In addition, this study aimed to highlight areas in need of improvement in the safety of patients.

The following questions were addressed in this investigation:

• What is the perception of nurses of PSC at a university hospital in Palestine?
• Is there a relationship between the outcome dimensions (frequency of events reported and overall perceptions of safety) and the other dimensions of PSC?
• What is the relationship between selected demographic variables (gender, age, tenure with the hospital, tenure in the work area, tenure in the profession, and hours worked per week) and nurses’ perceptions of PSC?

## Methods

### Study design

A cross-sectional study design with a self-reported survey through an online platform (SurveyMonkey®) was employed over two weeks of data collection. This study followed the STROBE (Strengthening the Reporting of Observational Studies in Epidemiology) guidelines for reporting cross-sectional studies.

### Setting

The study was carried out at NNUH, a non-profit and the only academic accredited hospital in Palestine that provides a wide range of health services. The hospital has 131 beds with almost full occupancy. It has been recently accredited by Joint Commission International (JCI) as the first and only accredited academic medical center in Palestine. The hospital has 267 nurses working in 18 inpatient and outpatient units.

### Sampling plan

The study population targeted nurses working at NNUH between June 25, 2021, to July 8, 2021. A convenience sample was applied to select the nurse’s staff. The inclusion criteria were: 1) nurses working in inpatient units and outpatient units, and 2) nurses having at least six months of working experience at this hospital. This ensured that all nurses who worked directly with patients could participate in the study, including head nurses. Exclusion criteria were: 1) aid nurses since they don’t work directly with patients and had been recently employed, 2) nurses who work in units that have no patients, like Central Sterilization Department, 3) nurses with experience of fewer than 6 months to guarantee that nurses are more directly involved with patient care, and 4) nurses on leave during the time of collecting the data.

### Instruments

The instrument used in the study had two parts. The demographic section was the first part which included questions on sex, age, number of working hours per week, and tenure in the profession, hospital, and unit. The second part included questions from the Arabic version of the Hospital Survey on Patient Safety Culture (HSOPSC). This version was translated by Najjar et al. [14]. To utilize the tool, a request and approval were sought. The HSOPSC is a survey tool to examine PSC from the perspective of hospital personnel [5]. The original Survey on Patient Safety Culture (SOPS®) Hospital Survey was established by the Agency for Healthcare Research and Quality (AHRQ) [15] and it was frequently used globally and in the Middle East area to investigate and analyze PSC in hospital settings, particularly from the nurse’s point of view. HSOPSC measured perception about safety culture, 42 items were used in 12 dimensions including “feedback about errors”, “communication openness”, “staffing”, “management support for patient safety”, “transitions and handovers”, “nonpunitive response to errors”, “organizational learning”, “supervisor/manager expectations”, “teamwork across units”, “teamwork within units”, “frequency of events reported” and “overall perception of safety”.

The survey used a Likert scale of 5 points (from 1 means ‘strongly disagree’ to 5 means ‘strongly agree’). Some items were represented as (from 1 means ‘always’ to 5 means ‘poor’. The score of all items in each dimension reflected the hospital's strengths and areas that need enhancements, this was obtained by calculating the frequency and percentage of positive responses for every dimension [(total # of positive responses/total # of respondents on each item) × 100%]. Combining answers of (strongly agree or agree or always and most of the time) and scoring about 75% and above illustrated the positive areas representing the strongly perceived dimensions. However, areas with a score of 50% or less were flagged as needing improvement [5].

The translated version of the survey was also used in Palestine, it has good psychometric properties, and the Cronbach’s coefficient alpha was “0.87” [14].

### Data collection

The survey was sent to all nurses using a SurveyMonkey® account by the quality office to their work emails. Another email was sent after one week of distributing the survey to ensure that there were no emails missed. The link sent contained an introduction with an overview of the study goals, outcomes, and instructions. The explanation also discussed voluntary participation and the freedom to withdraw at any time. collected data then was downloaded and kept in a safe-locked external hard drive to be analyzed.

### Data analysis

The data collected from the survey was organized, cleaned, and checked for errors using Microsoft Excel 2019, then coded, transferred, and analyzed by the Statistical Package for the Social Sciences (SPSS) v.26 software. The researcher used the AHRQ Hospital Survey on Patient Safety Culture Version 1.0: User's Guide [16] to help analyze the collected data. The survey data were analyzed using descriptive and inferential statistics. Descriptive statistics, frequency tables, and percentages were applied according to the level of analysis, and the results were displayed in tables. Univariate and multiple regression were used to examine the relationships. P-value < 0.05 considered statistically significant.

## Results

The survey was sent to a total of 240 nurses, and the overall response rate was 53% (*n* = 127). Twenty surveys were incomplete, so useful surveys used in the analysis were 107 (44.5%). Most of the nurses were Registered Nurses (RN) (95.3%, *n* = 102). The male nurses were 61.7% (*n* = 66) of the respondents, and female nurses 38.3% (*n* = 41). The age of the respondents ranged from 21 to 36 years; the mean age was 28 years (SD = 3.88). Regarding the length of time in the profession of nursing, most of the nurses surveyed had worked from 1 to 5 years (32.7%, *n* = 35), and from 6 to 10 years (41.1%, *n* = 44) (Table 1).

**Table 1:** Participant characteristics

Variables	Mean (SD)	Range	N	N%
**Gender**				
Male	-	-	66	61.7
Female	-	-	41	38.3
**Age**				
	28.16 (3.88)	22—36		
**Tenure in profession**				
Less than 1 year		6 m – 1y	18	16.8
1 to 5 years		1—5	35	32.7
6 to 10 years		6—10	44	41.1
11 to 15 years		11—15	10	9.3
**Tenure with hospital**				
Less than 1 year		6 m – 1y	19	17.8
1 to 5 years		1—5	37	34.6
6 to 10 years		6—10	51	47.7
**Tenure in the work area**				
Less than 1 year		6 m – 1y	26	24.3
1 to 5 years		1—5	54	50.5
6 to 10 years		6—10	27	25.2
**Hours worked per week**				
Less than 20 h			1	0.9
20–29 h			26	24.3
40–69 h			80	74.8
**Staff position**				
Registered Nurse (RN)			102	95.3
Practical Nurse (PN)			2	4.7

The majority of the participants (74.8%, *n* = 80) worked about 40 to 69 h a week, while almost a third of them worked about 20 to 29 h a week. On the other hand, nearly half of the nurses (50.5%, *n* = 54) had a tenure in the work area from 1 to 5 years, and almost the same percentage (47.7%, *n* = 51) of the nurses had a tenure with the hospital between 6 to 10 years, and thirty-seven of the nurses (34.6%, *n* = 37) had a tenure with the hospital from 1 to five years (Table 1).

### Nurses’ perceptions of patient safety culture

From the nurses’ perspective, the perception of the patient safety culture in NNUH was appraised by the composite frequency of each dimension and by verifying the strengths and areas that require improvement regarding patient safety issues. The study results revealed that the composite frequencies ranged between 22 and 87%, as shown in Table 2.

**Table 2:** Dimension item responses for patients’ safety culture

Item #	Dimensions	Positive Score (Strongly agree/Agree) (%)	Neither (%)	Negative score (strongly disagree/disagree) (%)	Average % of positive response
Dimension 1: Teamwork Within Units					**86%**
A1	People support one another in this unit	89.7	4.7	5.6	89.7
A3	When a lot of work needs to be done quickly, we work together as a team to get the work done	85.0	7.5	7.5	85.1
A4	In this unit, people treat each other with respect	80.4	10.3	9.3	80.4
A11	When one area in this unit gets really busy, others help out	89.6	3.8	6.6	89.6
Dimension 2: Supervisor/Manager Expectations & Actions Promoting Patient Safety					**59**
B1	My supervisor/manager says a good word when he/she sees a job done according to established patient safety procedures	79.4	15.0	5.6	79.4
B2	My supervisor/manager seriously considers staff suggestions for improving patient safety	91.6	7.5	0.9	91.6
B3	Whenever pressure builds up, my supervisor/manager wants us to work faster, even if it means taking shortcuts (R)^a^	15.0	21.5	63.6	63.6
B4	My supervisor/manager overlooks patient safety problems that happen over and over (R)	95.3	2.8	1.9	1.9
Dimension 3: Organizational Learning—Continuous Improvement					**87**
A6	We are actively doing things to improve patient safety	98.1	0.9	0.9	98.1
A9	Mistakes have led to positive changes here	77.6	16.8	5.6	77.6
A13	After we make changes to improve patient safety, we evaluate their effectiveness	85.0	12.1	2.8	85.1
Dimension 4: Management Support for Patient Safety					**69**
F1	Hospital management provides a work climate that promotes patient safety	78.5	15.9	5.6	78.5
F8	The actions of hospital management show that patient safety is a top priority	83.0	12.3	4.7	83.0
F9	Hospital management seems interested in patient safety only after an adverse event happens (R)	23.6	32.1	44.3	44.3
Dimension 5: Overall Perceptions of Safety					**64**
A10	It is just by chance that more serious mistakes don’t happen around here (R)	50.5	18.7	30.8	30.8
A15	Patient safety is never sacrificed to get more work done	72.0	9.3	18.7	72.0
A17	We have patient safety problems in this unit (R)	15.9	15.9	68.2	68.2
A18	Our procedures and systems are good at preventing errors from happening	86.0	10.3	3.7	86.0
Dimension 6: Feedback & Communication About Error					**83**
C1	We are given feedback about changes put into place based on event reports	72.9	22.4	4.7	72.9
C3	We are informed about errors that happen in this unit	87.9	11.2	0.9	87.9
C5	In this unit, we discuss ways to prevent errors from happening again	86.9	10.3	2.8	86.9
Dimension 7: Communication Openness					**52**
C2	Staff will freely speak up if they see something that may negatively affect patient care	76.6	15.9	7.5	76.6
C4	Staff feel free to question the decisions or actions of those with more authority	35.5	30.8	33.6	35.5
C6	Staff are afraid to ask questions when something does not seem right (R)	25.5	32.1	42.5	42.5
Dimension 8: Frequency of Events Reported					**76**
D1	When a mistake is made, but is caught and corrected before affecting the patient, how often is this reported?	75.7	16.8	7.5	75.7
D2	When a mistake is made, but has no potential to harm the patient, how often is this reported?	73.8	17.8	8.4	73.8
D3	When a mistake is made that could harm the patient, but does not, how often is this reported?	77.6	13.1	9.3	77.6
Dimension 9: Teamwork Across Units					**59**
F2	Hospital units do not coordinate well with each other (R)	24.3	35.5	40.2	40.2
F4	There is good cooperation among hospital units that need to work together	70.1	19.6	10.3	70.1
F6	It is often unpleasant to work with staff from other hospital units (R)	20.6	33.6	45.8	45.8
F10	Hospital units work well together to provide the best care for patients	80.4	14.0	5.6	80.4
Dimension 10: Staffing					**52**
A2	We have enough staff to handle the workload	67.3	10.3	22.4	67.3
A5	Staff in this unit work longer hours than is best for patient care (R)	35.5	28.0	36.4	36.5
A7	We use more agency/temporary staff than is best for patient care	30.8	22.4	46.7	30.8
A14	We work in "crisis mode" trying to do too much, too quickly	74.8	17.8	7.5	74.8
Dimension 11: Handoffs & Transitions					**53**
F3	Things “fall between the cracks” when transferring patients from one unit to another (R)	19.6	30.8	49.5	49.5
F5	Important patient care information is often lost during shift changes (R)	7.5	27.1	65.4	65.4
F7	Problems often occur in the exchange of information across hospital units (R)	29.5	36.2	34.3	34.3
F11	Shift changes are problematic for patients in this hospital (R)	13.1	22.4	64.5	64.5
Dimension 12: Nonpunitive Response to Error					**22**
A8	Staff feel like their mistakes are held against them (R)	48.6	26.2	25.2	25.2
A12	When an event is reported, it feels like the person is being written up, not the problem (R)	45.8	25.2	29.0	29.0
A16	Staff worry that mistakes they make are kept in their personnel file (R)	72.6	17.0	10.4	10.4

^a^* R* means the item score should be reversed

The highest composite frequency of patient safety perception scored for both organizational learning and continuous improvement (87%) and teamwork within units (86%). In addition, the secondary high composite frequency (83%) was positively scored for the feedback and communication about error, while 76% responded positively to the frequency of events reported.

On the contrary, the dimension with the lowest positive score was the nonpunitive response to error (22%). In contrast, other dimensions were considered areas for possible improvement to enhance patient safety in the hospital (see Table 2).

### The relationship between PSC dimensions

Multiple regression analysis revealed that the dimension of communication openness was a significant predictor of nurses' overall perceptions of safety. The nurses who perceived more communication openness (β = 0.257, *p* = 0.019), had more overall perceptions of safety. However, there was no association with the rest of the dimensions (*R* = 0.448; adjusted *R*^2^ = 0.118).

Furthermore, the results in Table 3 showed that the sixth dimension “feedback and communication about error” was a significant predictor of the frequency of events reported. Nurses who perceived more feedback and communication about error had a higher frequency of event reporting (β = 0.334, *p* = 0.005) (see Table 3).

**Table 3:** Multiple regression analysis of patient safety culture measures in overall patient safety grade and frequency of reported events

Variables	The overall perceptions of safety			Frequency of events reported		
	β	T-test	P*	β	T-test	P*
Dimension 1: Teamwork Within Units	0.071	0.540	0.591	0.042	0.344	0.732
Dimension 2: supervisor/Manager Expectations & Actions Promoting Patient Safety	-0.004	-0.038	0.970	-0.017	-0.158	0.875
Dimension 3: Organizational Learning—Continuous Improvement	0.097	0.783	0.436	0.205	1.771	0.080
Dimension 4: Management Support for Patient Safety	0.012	0.106	0.916	-0.149	-1.465	0.146
Dimension 6: Feedback & Communication About Error	0.097	-0.849	0.398	0.334	2.877	0.005*
Dimension 7: Communication Openness	0.257	2.388	0.019*	0.024	0.239	0.812
Dimension 9: Teamwork Across Units	-0.067	-0.613	0.541	0.120	1.173	0.244
Dimension 10: Staffing	0.043	0.409	0.683	0.011	0.108	0.914
Dimension 11: Handoffs & Transitions	-0.083	-0.698	0.487	0.143	1.292	0.200
Dimension 12: Nonpunitive Response to Error	0.092	0.772	0.442	-0.04	-0.036	0.971

^*^Significance level *p* < 0.05

### Relationship between selected demographic data and PSC

The result of the multiple regression analysis in Table 4 revealed that there was no relationship between sex, hospital tenure, work area tenure, profession tenure, and hours worked per week with the PSC. While age was found to be a predictor of PSC (*p* = 0.046). However, when we ran a univariate regression to see the relationship with each variable independently, the regression revealed no relation with any of these variables. This indicated that none of those variables alone had a significant effect on PSC.

**Table 4:** The influence of demographics on the total score of PSC

Variables	Total Score of PSC		
	β	T-test	P*
Gender	-.118	-1.190	.237
Age	.296	2.017	.046 *
Tenure with Hospital	-.264	-1.358	.177
Tenure in the work area	-.163	-1.089	.279
Hours worked per week	-.017	-.171	.865
Tenure in Profession	.076	.396	.693

^*^Significance at *p* < 0.05

In summary, according to the regression analysis, we failed to reject the null hypothesis that there is no relationship between the dimension of “overall perceptions of safety” and other dimensions of PSC and among demographics with the total score of PSC. Furthermore, the null hypothesis that there is no relationship between the dimension of frequency of event reported and the other dimensions of the PSC was partially rejected based on a significant relationship with the dimension of feedback and communication about error (*p* = 0.005). Finally, the third null hypothesis that there is no relationship between the dimension of the overall perceptions of safety and the other PSC dimensions was partially rejected based on a significant relationship with the dimension of communication openness (*p* = 0.019).

## Discussion

This study was set up to assess the perception of PSC at NNUH. This study had shown that organizational learning and continuous improvement, teamwork within units, feedback and communication about error, and frequency of events reported were the most positive responses among the item frequency dimension of patient safety culture. The highest total response was the continuous improvement of organizational learning of NNUH at 87%. This was congruent with the result of similar studies [17–19]. This could be related to the high values of the nursing administration and fostering the nurses’ education and development. NNUH had developed a continuous nursing education department that provides training and evidence-based practice to nurses simply and competitively. Another explanation might be the hospital environment that supports organizational learning skills.

The second high dimension was teamwork within units of 86% score, which was similar to the results from previous studies [19–24]. Nurses in this hospital believe that good teamwork is crucial for improving patient safety culture. They perceived that hospital administration encourages good teamwork across hospital units and staff. Feedback and communication about error dimension was 83%, similar to previous studies [2, 18, 25]. This dimension was closely related to the event reporting dimension of 76%. The positive score on this dimension might be related to the fact that this hospital holds monthly meetings to discuss various reports and improvements per unit. The frequency of events reported dimension positive score was similar to previous related studies [26, 27]. The frequency of events reported can contribute continuously to learning from mistakes. Incident reports of safety issues make it possible to find possible causes of failures in work areas and structures, to tackle them to improve patient safety [21].

Another important finding was that this study's nonpunitive response to error was the least positive score of the dimensions. Previous related studies also reported this finding [18, 20, 22, 26, 28–31], which is a serious threat to patient safety related to staff fears of reporting events. Therefore, hospital administration must take serious actions regarding the items under the dimension of nonpunitive response to error to improve the culture of incident reporting and improve patient safety after all.

About the question of whether there is a relationship between the outcome dimensions (frequency of events reported, and overall perceptions of safety) and the other dimensions of PSC. This study using the regression model found that communication openness was a significant predictor of the overall perceptions of safety. This result reflected the result of Ree & Wiig's study [32], which found that communication openness was a positive predictor at 42% of the explained variance in the overall perceptions of safety. This was also similar to another study which found that communication openness was significantly one of the predictor dimensions of the overall perceptions of safety [32].

Another important finding from the regression model was that the dimension of feedback and communication about error dimension was a significant predictor of the frequency of events reported. Such culture will lead to disclosing any event or accident that may harm patient safety. This was similar to studies conducted in Saudi Arabia and Oman which found that feedback and communication about error was a predictor of the frequency of events reported [33, 34].

The current study results also showed that in demographic characteristics regarding the length of time in the nursing profession, most of the nurses surveyed had worked from 1 to 5 years (32.7%, *n* = 35), and from 6 to 10 years (41.1%, n = 44). The regression analysis showed that there was no significant prediction of years of experience. These results were in away with the findings of the study done by Khater et al. [26] pointed to nurses’ experience ranging between 1 and 26 years (M = 7.5 years, SD = 5.377). The regression analysis revealed that the higher the total years of experience, the better the nurses’ perception of patient safety culture.

Moreover, the result of regression analysis in our study revealed that there was no relationship between sex, hospital tenure, work area tenure, profession tenure, hours worked per week, and the total score of patient safety culture. This was congruent with the results of Ammouri et al. [27] who found that demographic characteristics of the participants such as gender, educational degree, hospital position, and unit of work did not have a significant relationship with the nurses’ perception of patient safety culture. In contrast, we found that age was a predictor of the perception of PSC. This finding was consistent with Khater et al. [26], who found that older respondents had a lower perception of patient safety culture than younger nurses; however, this difference was not highly significant (*p* = 0.048). This was also similar to the results of other studies which found a negative relationship between age and the perception of PSC [35, 36].

The results of our study on the relationship with selected demographic characteristics with a total score of PSC indicated that the perception of nurses toward PSC was genuine and not affected by other selected factors. This will help administrators to work on improving PSC easily.

Furthermore, in Jordanian hospitals, the working shift system was based on 12 h per shift rather than the traditional 8 h per shift. This could lead to a decrease in alertness, a decrease in productivity, an increase in staff fatigue, and an increase in medical errors [26]. Although this finding contradicted our study in NNUH which is a teaching hospital, and the working shift system there was based on 8 h per shift, we found that there was no significant relationship between PSC and weekly working hours.

### Study limitations

This study was conducted in one hospital, and the response rate was low; this may be due to the method used for distributing the survey through work emails. The staff might not look into their emails, resulting in a low response rate. Additionally, the participants in this study were nurses; therefore, the results only reflected only the perception of nurses. There was a need to assess the patient safety culture from other healthcare providers’ perspectives in this hospital, such as physicians, technicians, etc. Thus, the findings of this study cannot be generalized, and further studies to compare the results with other hospitals in the country are recommended. Furthermore, because our study was designed as a cross-sectional survey, we could not determine causality between study factors.

## Conclusions

This study provided a general assessment of perceived safety among nurses in a hospital. Nurses perceived the overall patient safety culture positively; organizational learning, teamwork within units, feedback and communication about error, and frequency of events reported were the most positive areas. However, we found that nurses negatively perceived a nonpunitive response to error. Therefore, strenuous efforts are required by hospital management to improve the culture toward incident reporting, including “but not limited to” increasing the awareness of the importance of incident reporting, engaging staff members in the corrective actions of incidents reported, educating staff, and blaming the process rather than blaming the individuals.

## Data Availability

The data sets supporting the results of the current research are available from the corresponding authors upon request.

## Abbreviations

PSC: Patient Safety Culture
IRB: Institutional Review Board
SPSS: Statistical Software Package for the Social Sciences
Q1: First quartile
Q3: Third quartile
SD: Standard deviation
HSOPSC: Hospital Survey of Patient Safety Culture
JCIA: Joint Commission International Accreditation
PSFHI: Patient Safety Friendly Hospital Initiative
IPSG’s: International Patient Safety Goals
NNUH: An-Najah National University Hospital
JCI: Joint Commission International
AHRQ: Agency for Healthcare Research and Quality
RN: Registered Nurses
PN: Practical Nurse
SOPS®: Surveys on Patient Safety Culture
